# Supplementary material for: An ancient germ cell-specific RNA-binding protein protects the germline from cryptic splice site poisoning
Source: eLife. 2019 Jan 24;8:e39304. doi: 10.7554/eLife.39304 (PMC6345566; doi:10.7554/eLife.39304)
Supplement: Figure 3—source data 7. — Summary of GO terms and MGI phenotypes of genes that are regulated at the splicing level by RBMXL2 (compiled from Figure 3—source datas 5 and 6). [file elife-39304-fig3-data7.docx]

| GO term Spermatogenesis | **GO term GO term Germ MGI Meiotic cell cell infertility cycle process development phenotype** | | | **MGI lethal MGI phenotype development**  **phenotype** | |
| --- | --- | --- | --- | --- | --- |
| **Brca2** | Brca2 | Brca2 | Kdm4d | Ppox | Ppp3ca |
| **Catsperb** |  | Catsperb | Ccdc62 | Mylk | Ttc28 |
| **Hmga2** | Hmga2 | Pcsk4 | Hmga2 | Bcl11b | Clec16a |
| **Odf2** | 4930447C04R | 4930447C04Ri | Alms1 | Zfp148 | Alms1 |
|  | ik | k |  |  |  |
| **Tlk2** | Mlh3 | Alms1 | Meioc | Ald5h1 | Plec |
| **Pcsk4** |  |  | Gba2 | a | Tmpo |
| **Lrguk** |  |  | Brca2 | Gabpb1 | Nxn |
| **4930447C04Rik** |  |  | Poln | Clec16a | Med1 |
| **Slc9c1** |  |  | Pcsk4 | Tln1 | Tln1 |
| **Alms1** |  |  | Zbtb16 | Brca2 | Brca2 |
| Zfp148 | | | | Ebp41 | Vit |
| Mlh3 | | | | Tnrc6a | Slc39a13 |
| Gabpa | | | | Furin | Furin |
| Nectin3 | | | | Wdpcp | Wdpcp |
| Slc9c1 Gabpa | | | | | |
| Lnpk Slc39a8 | | | | | |
| Tmed10 Med1 | | | | | |
| Clip1 Slc7a1 | | | | | |
| Brd4 Lnpk | | | | | |
| Hip1 Psmc3 | | | | | |
| Lrrcc1 Tmed10 | | | | | |
| Myo7a Coq2 | | | | | |
| Odf2 Brd4 | | | | | |
| Spag6l Hip1 | | | | | |
| 4930447C04R Nxn | | | | | |
| ik | | | | | |
| Dnal1 | | | | | |
| Seh1l | | | | | |
| Plec | | | | | |
| Mapk25 | | | | | |
| Atp8b2 | | | | | |
| Elac2 | | | | | |
| Cep67 | | | | | |

Figure 3 - figure supplement 2
